# Clinical practice guideline for blood‐based biomarkers in the diagnostic workup of Alzheimer’s disease within specialized care settings: A report from the Alzheimer's Association

**DOI:** 10.1002/alz70861_108894

**Published:** 2025-12-23

**Authors:** Sebastian Palmqvist, Heather Whitson, Laura A Allen, Maria C. Carrillo, Rebecca M. Edelmayer, Douglas R. Galasko, Oskar Hansson, Lara Kahaleh, Thomas K Karikari, Simin Mahinrad, Mary Beth McAteer, Hamid Okhravi, Madeline Paczynski, Sarah Pahlke, Suzanne E. Schindler, Marc Suárez‐Calvet, Charlotte E. Teunissen, Henrik Zetterberg, Malavika Tampi

**Affiliations:** ^1^ Clinical Memory Research Unit, Department of Clinical Sciences Malmö, Faculty of Medicine, Lund University, Lund Sweden; ^2^ Memory Clinic, Skåne University Hospital, Malmö Sweden; ^3^ Duke University, Durham, NC USA; ^4^ Mayo Clinic, Rochester, MN USA; ^5^ Medical & Scientific Relations Division, Alzheimer's Association, Chicago, IL USA; ^6^ Alzheimer's Association, Chicago, IL USA; ^7^ University of California, San Diego, La Jolla, CA USA; ^8^ Clinical Memory Research Unit, Department of Clinical Sciences Malmö, Faculty of Medicine, Lund University, Sweden, Lund Sweden; ^9^ Infectious Diseases Society of America, Arlington, VA USA; ^10^ University of Pittsburgh, Pittsburgh, PA USA; ^11^ Virginia Mason Franciscan Health, Seattle, WA USA; ^12^ Eastern Virginia Medical School, Norfolk, VA USA; ^13^ Washington University School of Medicine, St. Louis, MO USA; ^14^ Barcelonaβeta Brain Research Center (BBRC), Barcelona Spain; ^15^ Amsterdam University Medical Centers, Amsterdam Netherlands; ^16^ Amsterdam University Medical Centre, Amsterdam Netherlands; ^17^ University of Gothenburg, Mölndal Sweden

## Abstract

**Background:**

In recent years, blood‐based biomarkers (BBMs) have transformed the diagnostic landscape of Alzheimer’s disease (AD), with some now approaching readiness for clinical implementation. This progress aligns with the growing importance of accurate early diagnostics and availability of anti‐Aβ therapies for the treatment of early symptomatic AD, reinforcing the need for more rapid and early diagnostic capabilities. To address this need, the Alzheimer’s Association convened a multidisciplinary panel of clinical experts, subject‐matter specialists, and guideline methodologists to conduct a systematic review and develop evidence‐based recommendations for the use of BBMs in the diagnostic evaluation of AD. The scope of this guideline is focused on individuals with cognitive impairment ‐ either MCI or dementia ‐ who are undergoing diagnostic assessment in secondary or tertiary care settings.

**Method:**

The panel conducted a systematic review to assess BBMs' diagnostic test accuracy in detecting amyloid pathology for triaging (≥90% sensitivity, ≥75% specificity) and confirmatory (≥90% sensitivity and specificity) diagnostic workup. The BBMs of interest included plasma *p* ‐tau and Aβ tests measuring the following analytes: *p* ‐tau217, %*p*‐tau217, *p* ‐tau181, *p* ‐tau231, and Aβ42/Aβ40 ratio. The reference standard tests included CSF, amyloid PET, or neuropathology examination. The panel applied the GRADE approach to assess the certainty of the evidence and the GRADE Evidence‐to‐Decision Framework to develop its recommendations.

**Result:**

Across 49 observational studies meeting eligibility criteria, 31 different BBM tests were evaluated. Using predefined decision thresholds, the panel determined whether each test has 1) sufficient diagnostic test accuracy to be used as a triaging test where a positive test is to be confirmed by PET or CSF, 2) sufficient diagnostic test accuracy as a confirmatory test to replace PET or CSF, or 3) insufficient diagnostic test accuracy to recommend current use in clinical practice. Recommendations will be provided in case any BBMs met a priori DTA thresholds.

**Conclusion:**

BBMs can improve early AD diagnosis and expand access to disease‐modifying therapies. Evidence‐based guidelines are key to standardizing their use and will be updated as new evidence and applications emerge.

